# A rare case of tuberculosis-induced hypercalcemia

**DOI:** 10.11613/BM.2020.030801

**Published:** 2020-08-05

**Authors:** Loris Wauthier, Xavier Theunssens, Patrick Durez, Catherine Fillée, Diane Maisin, Damien Gruson

**Affiliations:** 1 Department of Clinical Biochemistry, Cliniques Universitaires St-Luc and Université Catholique de Louvain, Brussels, Belgium; 2 Department of Rheumatology, Cliniques Universitaires St-Luc and Université Catholique de Louvain, Brussels, Belgium; 3 Pôle de Pathologies rhumatismales, Institut de Recherche Expérimentale et Clinique, Université Catholique de Louvain, Brussels, Belgium; 4 Pôle de recherche en Endocrinologie, Diabète et Nutrition, Institut de Recherche Expérimentale et Clinique, Cliniques Universitaires St-Luc and Université Catholique de Louvain, Brussels, Belgium

**Keywords:** hypercalcemia, tuberculosis, rheumatoid polyarthritis, calcitriol

## Abstract

Laboratory investigations of hypercalcemia involve testing of various biochemical parameters such as parathyroid hormone (PTH), 25-(OH) Vitamin D (25-(OH) VitD), 1,25-(OH)_2_ Vitamin D3 (calcitriol) and PTH related peptide (PTHrp). We herein present an atypical case of severe hypercalcemia in a patient with rheumatoid arthritis who has been treated for years by various biological disease-modifying antirheumatic drugs (DMARDs) and suddenly presented with general state alteration, oedema and ulceration of her right ankle. We illustrate how tuberculosis (TB) can cause high calcitriol concentration and subsequently lead to potentially severe hypercalcemia. Moreover, we highlight the importance of TB testing and follow-up in patients treated with biological DMARDs.

## Introduction

From the laboratory perspective, investigations in the course of de novo or chronic hypercalcemia require the use of various assays for the differential diagnosis between primary hyperparathyroidism, multiple myeloma, familial hypercalcemia, malignancy, Vitamin D (VitD) intoxication or granulomatosis. The most frequently used tests for this workup besides calcemia are: parathyroid hormone (PTH), PTH related peptide (PTHrp), 25-(OH) Vitamin D (25-(OH) VitD), and 1,25-(OH)_2_ Vitamin D3 (calcitriol) (*1*, *2*).

Tuberculosis (TB) in its pulmonary and disseminated forms is known to be a rare but well-described cause of hypercalcemia and is described to be associated with high concentrations of calcitriol (*3*–*5*). Here, we report the case of hypercalcemia in a patient with disseminated TB and articular presentation, in the context of long-term treatment of rheumatoid arthritis (RA) by biological disease-modifying antirheumatic drugs (DMARDs).

## Case presentation

The patient was a 67-year-old Caucasian female who has been consulting in the rheumatology department at our facility in the course of an erosive seropositive RA, which was poorly evolving. Among other medications, the patient was treated for several years with different DMARDs, such as etanercept and more recently with infliximab and then tocilizumab, together with methotrexate. At her last outpatient visit, hospitalization was planned as her inflammatory disease was worsening and associated with general state alteration, fever, mild dyspnoea as well as seemingly chronic oedema and ulceration of her right ankle articulation. Her routine blood examination, from which relevant biochemistry results are summarized in Table 1, showed increased C-reactive protein concentrations (CRP) to 114.2 mg/L (upper reference limit (URL): 5 mg/L), concordant with her active inflammatory pathology, acute renal failure (creatinine (CREA): 234 µmol/L (reference range (RR): 53 - 115 µmol/L), urea: 43.9 mmol/L (RR: 5.4–17.9 mmol/L), estimated glomerular filtration rate (eGFR) based on the chronic kidney disease epidemiology collaboration (CKD-EPI) equation: 18 mL/min/1.73m^2^ (lower reference limit: 60 mL/min/1.73m^2^) and open hypercalcemia (total calcium (Ca) concentration: 4.38 mmol/L (RR: 2.20–2.55 mmol/L), confirmed with a clearly increased albumin-corrected Ca of 4.43 mmol/L (RR: 2.20–2.55 mmol/L)). This led to anticipated hospitalization. The patient signed an informed consent form for anonymous publication of medical data.

**Table 1:** Relevant biochemistry results at time of hospitalization

**Parameter**	**Observed concentration**	**Reference range**
CRP (mg/L)	114.2	< 5
Urea (mmol/L)	43.9	5.4-17.9
CREA (µmol/L)	234.3	53-115
eGFR (CKD-EPI) (mL/min/1.73m^2^)	18	> 60
Sodium (mmol/L)	137	135-145
Potassium (mmol/L)	3.96	3.5-5
Phosphate (mmol/L)	1.76	0.81-1.45
Total calcium (mmol/L)	4.38	2.20-2.55
Albumin-corrected calcium (mmol/L)	4.43	2.20-2.55
Albumin (g/L)	38	35-52
Intact PTH (ng/L)	14	15-80
PTHrp (ng/L)	< 20	< 20
25-(OH) VitD (nmol/L)	87.5	75-250
1,25-(OH)_2_ Vitamin D3 (pg/mL)	171	20-79
CRP – C-reactive protein. CREA – creatinine. eGFR – estimated glomerular filtration rate. CKD-EPI – chronic kidney disease epidemiology collaboration equation. PTH – parathyroid hormone. PTHrp – PTH related peptide. 25-(OH) VitD – 25-(OH) vitamin D.		

## Clinical and laboratory investigations

The clinician’s investigations included first suspicion of multiple myeloma as age, renal function and calcemia were suggestive of such pathology. However, neither serum protein electrophoresis nor immunoglobulins and free light chains assay nor urine immunofixation showed the presence of monoclonal immunoglobulin. Urinalysis exhibited no significant features.

Besides, as intact PTH concentration was 14 ng/L (RR: 15–80 ng/L), hyperparathyroidism and familial hypercalcemia were excluded. PTHrp concentration was < 20 ng/L (URL: 20 ng/L), excluding a neoplastic origin for the high Ca concentration observed.

Notably, 25-(OH) VitD concentration was 88 nmol/L (RR: 75–250 nmol/L) but calcitriol concentration was interestingly high (171 pg/mL (RR: 20–79 pg/mL), suggesting pathologies such as lymphoma or granulomatous diseases such as sarcoidosis and tuberculosis. Various assays were used to perform differential diagnosis. Urine Ca normalized for urine CREA was 2.26 mM/mM (URL: 0.40 mM/mM) and showed hypercalciuria. Angiotensin converting enzyme activity was 68 U/L (RR: 12–68 U/L) and blood lysozyme was 131.4 mg/L (RR: 9.6 –17.1 mg/L) (increased around 8 times the upper limit of the reference range).

The final diagnosis was achieved by performing a further examination of the patient’s right ankle. Radiography and magnetic resonance imaging showed active multifocal osteoarthritis while positron emission tomography-computed tomography showed multiple hypermetabolic lesions at the pericardial, periarticular, muscular, meningeal, and lymph nodes hypermetabolic lesions and also revealed wide pulmonary cavities. Microbiological samples were collected as surgery was performed and were sent to our bacteriology laboratory for culture and Koch’s bacillus research. Microscopic examination of auramine-stained slides showed the presence of acid-fast bacilli, which was also detected by a semi-quantitative nested polymerase chain reaction. Culture on liquid medium furthermore confirmed positivity for *Mycobacterium tuberculosis* complex and further identification of *Mycobacterium bovis* was performed by Sciensano (National Reference Center for the diagnosis of tuberculosis and human mycobacterioses, Brussels, Belgium)

## Treatment and outcome

Hypercalcemia and acute renal failure were treated in the emergency room by the administration of large amounts of sodium chloride, 40 mg furosemide, and 90 mg pamidronate. This temporarily decreased albumin-corrected Ca concentration to 2.80 mmol/L, which nonetheless stayed above our institution’s reference range (2.20–2.55 mmol/L) for days after the patient was taken care of. Tuberculosis was treated by classical 4-drugs therapy (rifampin, isoniazid, pyrazinamide, ethambutol + pyridoxine) until antibiogram and formal identification suggested stopping pyrazinamide because of resistance.

Normalization of eGFR to 63 mL/min/1.73m^2^ was not achieved before one week after antibiotic treatment onset and total Ca concentration to 2.35 mmol/L (RR: 2.20–2.55 mmol/L) (no albumin-corrected value available at that time point) was documented one week later. Ulceration and oedema of the ankle also started healing at the same time, initiating a slow recovery of the wound.

Usual RA treatment was suspended and limited to daily 7.5 mg prednisolone. Tocilizumab treatment was planned to be resumed when infectious symptoms would be under control.

## Discussion

Hereby, we described an unusual case of hypercalcemia in the context of disseminated TB in a patient with RA treated by biological DMARDs. The patient displayed increased calcitriol concentration and normal 25-(OH) VitD whereas PTH concentration was decreased and PTHrp was within the reference range. The diagnosis of TB was later confirmed by clinical and microbiological investigations and concluded to mycobacterial infection.

Biochemical results were consistent with previous reports of such cases of TB-induced hypercalcemia (*6*, *7*). In this particular context of TB-induced hypercalcemia, a first hypothesis for the Ca raise could be the high calcitriol concentrations, leading to an increased intestinal Ca absorption and osteoclastic bone resorption (*3*–*5*). Such an increase in calcitriol concentrations can be linked to a triggered 1-α-hydroxylase activity of the macrophages within the granulomatous reaction sites of patients presenting TB or sarcoidosis (*8*).

Additionally, another interesting hypothesis could be the activation/reactivation of TB, and its related impact on Ca concentrations, in the context of RA, handled by long-term anti-TNFα and then IL-6 antagonist treatment. The mechanism of action of both treatments target inflammatory cytokines and therefore are well-described causes of latent TB reactivation or primary TB infection (*9*-*11*). These cytokines are indeed involved in the immuno-inflammatory response to mycobacterial infections (*12*, *13*). This major adverse effect of these biological DMARDs is known to be higher with etanercept than with other treatments and is furthermore not only described in TB but also in nontuberculous mycobacteria infections (*14*, *15*).

Our study has several limitations. A first one is the lack of measurement of an ionized-Ca assay, a reference method to confirm hypercalcemia that displays higher sensitivity and specificity than total Ca (*2*). However, the hypercalcemia of our case was confirmed by albumin-corrected Ca. A second limitation could be the start of the biological DMARDs treatment in another care setting, unknown to the authors, misleading the information of an initial screening for TB in our patient in this facility.

This case is yet another proof of the importance of TB screening and follow-up in patients treated with anti-TNFα and non-anti-TNFα treatments.

In conclusion, this case illustrates how granulomatosis and by extension, TB, can cause potentially severe hypercalcemia. This was described to be associated with a granulomatosis-triggered increase in calcitriol concentration. Moreover, we highlight the importance of TB monitoring in patients treated by anti-TNFα or non-anti-TNF biological DMARDs in order to prevent infection or reactivation.
